# Connectional neuroanatomy of U-fibers in the rhesus monkey brain

**DOI:** 10.64898/2026.06.06.730597

**Published:** 2026-06-15

**Authors:** Tyler J. Capen, Lauren J. O’Donnell, Fan Zhang, Edward H. Yeterian, Yogesh Rathi, Nikos Makris, Douglas L. Rosene, R. Jarrett Rushmore

## Abstract

The superficial white matter (SWM), the region of white matter immediately beneath the gray matter-white matter (GM-WM) border, contains short cortico-cortical association fibers that interconnect neighboring cortical regions. The SWM is estimated to comprise the majority of axons in the cerebral white matter and is thus thought to play a major role in cortical information processing. Despite this prominence, and growing attention in the diffusion MRI (dMRI) field, the connectional organization of the SWM remains poorly defined. A major component of the SWM is U-shaped fiber bundles, classically depicted in brain dissections as bundles beneath sulci that interconnect adjacent gyri. The prevailing view, from both brain dissection and dMRI, considers U-fibers to be ubiquitous elements of the SWM that form symmetric connections between corresponding portions of adjacent gyri beneath all sulci, and they are routinely reconstructed in tractography as a layer of short association connections. However, their actual prevalence and organization in the primate brain have never been systematically evaluated with experimental tract tracing. To address this gap, we analyzed 28 archival macaque tract-tracing cases in which anterograde tracers were injected on gyri adjacent to major cortical sulci (intraparietal, central, principal, superior temporal), all of which have clear anatomical correspondences to sulci in the human cerebral cortex. Several organizational principles emerged. First, although short association fibers were present in every case, U-fibers - operationally defined as labeled fibers that left the injection site, passed beneath a sulcus, and terminated on the adjacent gyrus - were only observed in a minority of cases. Second, U-fiber incidence was sulcus-dependent: U-fibers were consistently present beneath the principal and central sulci, but uncommon beneath the intraparietal and superior temporal sulci. Third, when present, U-fibers did not follow a uniform trajectory - some took a course symmetric and orthogonal to the sulcus, whereas others followed oblique trajectories to more distant regions. In addition to U-fibers, we identified shorter association fiber bundles that terminated on the sulcal bank proximal to the injection, or on the opposing bank after they crossed the fundus of the sulcus, evidence that the SWM contains multiple classes of short association fiber bundles rather than a single canonical U-fiber system. We further identified a consistent 200-300 µm band of white matter beneath cortical layer 6 that contained exclusively short association fibers on approach to their cortical terminations and was selectively avoided by deeper long cortico-cortical pathways. This band was present across all examined sulci, suggesting that it is a conserved feature of the cortical white matter. Notably, U-fibers were found both within this band and in the white matter below. We also identified deeper fiber bundles that adopted a U-shaped course beneath a sulcus but did not interconnect adjacent gyri. Thus, a U-shaped trajectory alone does not define a U-fiber. Together, these findings constitute the first systematic connectional study of cortical U-fibers in major sulci of the non-human primate brain and indicate that the white matter beneath the cortex is a highly complex system in which short association fiber bundle organization reflects the selective connectivity of the cortical areas separated by a sulcus, rather than a uniform U-fiber architecture imposed by the geometry of the overlying sulcus. These results have potential consequences for dMRI and anatomical studies of white matter and provide an anatomically grounded basis to better define SWM organization in anatomy and tractography.

## Full Text Availability

The license terms selected by the author(s) for this preprint version do not permit archiving in PMC. The full text is available from the preprint server.

